# Decoding signaling crosstalk in pulpitis: pathogenesis and precision therapeutics

**DOI:** 10.3389/fcell.2026.1872666

**Published:** 2026-06-17

**Authors:** Zhaohui Jia, Zongzong Sun, Hairui Zhou

**Affiliations:** 1 The First Affiliated Hospital, College of Clinical Medicine, Henan University of Science and Technology, Luoyang, Henan, China; 2 The Third Affiliated Hospital of Zhengzhou University, Zhengzhou, Henan, China; 3 Key Laboratory of Microecology-immune Regulatory Network and Related Diseases School of Basic Medicine, Jiamusi University, Jiamusi, Heilongjiang, China

**Keywords:** pulpitis, research progress, responsive biomaterials, signaling crosstalk, vital pulp therapy

## Abstract

Irreversible pulpitis presents a complex pathological challenge, marked by severe inflammation within the low-compliance dentin chamber. Its progression to pulpal necrosis is governed by intricate molecular interactions. Hyperactivation of the TLR/NF-κB and MAPK pathways triggers a pro-inflammatory cascade, while HIF-1α accumulation under hypoxic conditions induces significant metabolic reprogramming. Meanwhile, the NLRP3 inflammasome exacerbates tissue damage by promoting programmed cell death. Endogenous Wnt/β-catenin and TGF-β/Smad signaling pathways strive to promote structural repair and dentinogenesis, but are often suppressed by the hyperactive inflammatory environment. Recent translational research highlights the potential of targeted molecular interventions to address this pathological imbalance. This review synthesizes emerging therapeutic strategies aimed at these key pathways, emphasizing the pharmacological use of specific phytochemicals, epigenetic regulators, and specialized pro-resolving mediators, particularly Resolvin E1, which collaboratively attenuate NF-κB-driven inflammation while enhancing Wnt- and TGF-β-mediated regenerative processes. Additionally, this review discusses the development of next-generation, microenvironment-responsive biomaterials designed to adapt dynamically to pulpal hypoxia and oxidative stress. Ultimately, understanding these signaling interactions lays a molecular foundation for advancing objective, biomarker-based diagnostics and precision therapeutics, offering promising prospects for predictable vital pulp therapy.

## 1 Introduction

Dental caries and physical trauma often compromise the structural integrity of enamel and dentin, exposing the underlying pulp to microbial invasion and triggering pulpitis (Farges et al., 2015; Kakehashi et al., 1965; Ricucci et al., 2014). Traditionally, symptomatic irreversible pulpitis has been treated with root canal therapy (RCT), which frequently leads to the loss of tooth vitality, disruption of intrinsic immune surveillance, and reduced long-term biomechanical resilience (Duncan et al., 2019; Tang et al., 2010). Consequently, modern endodontics has shifted toward minimally invasive vital pulp therapy (VPT) and regenerative approaches (Duncan et al., 2019; Schwendicke et al., 2016). However, the clinical success of VPT hinges on the accurate assessment of the pulpal inflammatory status and the precise pharmacological modulation of the pathological microenvironment (Cooper et al., 2017; Rechenberg et al., 2016).

The dental pulp is encased within a rigid, low-compliance dentin chamber (Bai et al., 2025; Korkmaz et al., 2025; Smaida et al., 2025). Upon pathogenic insult, inflammatory exudate accumulates rapidly in this confined space, resulting in interstitial hypertension, severe hypoxia, and profound metabolic stress (Miao et al., 2026; Shao et al., 2025). Within this spatially constrained environment, the fate of the pulpal tissue—whether progressing to irreversible necrosis or shifting toward reparative dentinogenesis—depends not on a linear sequence of events but on a dynamic and interconnected network of intracellular signaling pathways. Specifically, pathogen-associated molecular patterns (PAMPs) initiate the pro-inflammatory response primarily via the Toll-like receptor (TLR)/nuclear factor kappa B (NF-κB) and mitogen-activated protein kinase (MAPK) signaling axes. Conversely, ischemic stress from vascular compression hyperactivates hypoxia-inducible factor-1α (HIF-1α)-mediated metabolic reprogramming and NLRP3 inflammasome-driven pyroptosis. Simultaneously, endogenous reparative pathways, such as Wnt/β-catenin and transforming growth factor-beta (TGF-β)/Smad signaling, counteract inflammation and promote tissue repair and remodeling. Therefore, the interplay between pro-inflammatory and reparative signaling networks dictates the progression and reversibility of pulpitis.

Recognizing these signaling pathways as critical therapeutic targets, contemporary strategies have evolved beyond passive capping materials to active, pathway-selective interventions. Growing evidence supports the efficacy of natural bioactive compounds, epigenetic regulators like non-coding RNAs, and endogenous pro-resolving lipid mediators in selectively modulating pro-inflammatory cascades and inhibiting programmed cell death. In parallel, advances in smart biomaterials, including bioactive calcium silicate cements and microenvironment-responsive nanocomposite hydrogels, facilitate localized, sustained, and stimulus-triggered delivery of signaling modulators (Zhang L. et al., 2025; Zhang Q. et al., 2025). Previous studies have shown that selectively suppressing key inflammatory nodes such as NF-κB and NLRP3 (Wang Y. et al., 2024), or enhancing reparative pathways like Wnt/β-catenin and TGF-β/Smad, can effectively resolve pathological antagonism and restore pulpal homeostasis (Nam et al., 2026).

Despite significant advances in molecular understanding, a comprehensive, mechanism-driven synthesis connecting the spatiotemporal progression of pulpitis with pathway-targeted therapeutics is still lacking. This review aims to provide a mechanistically grounded overview of the molecular landscape governing pulpal inflammation and regeneration.

## 2 Core signaling pathway network regulation in pulpitis pathogenesis

**FIGURE 1:**
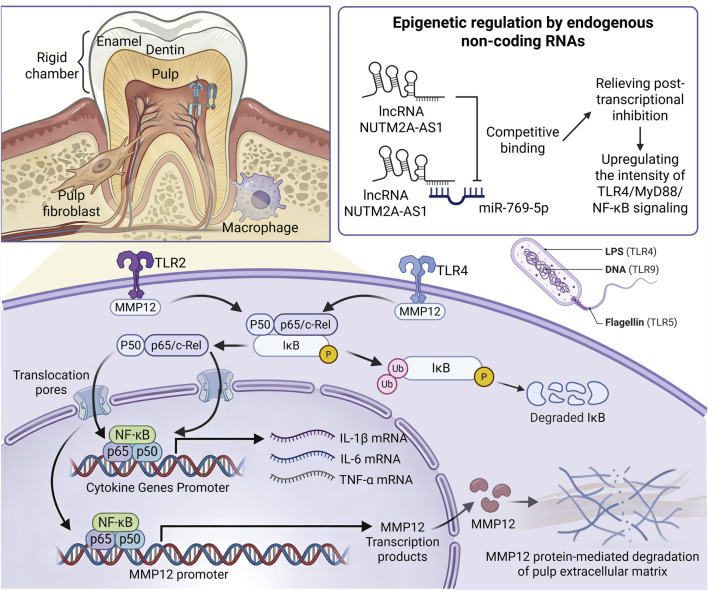
Molecular mechanisms of pathogen recognition and pro-inflammatory cascade amplification in the early stage of pulpitis. During initial infection, lipopolysaccharide (LPS) from Gram-negative bacteria binds to the transmembrane receptors TLR2 and TLR4 on dental pulp cells (fibroblasts/macrophages), recruiting the adaptor protein MyD88. This initiates a kinase cascade leading to the phosphorylation and ubiquitin-mediated degradation of IκB, thereby releasing the NF-κB (p65/p50) dimer for nuclear translocation. Epigenetically, the lncRNA NUTM2A-AS1 acts as a competitive endogenous RNA, relieving the post-transcriptional inhibition mediated by miR-769–5p and further amplifying this pathway. Concurrently, physical and chemical stresses activate the P2X3 receptor, which drives downstream signaling amplification via the MAPK cascade (p38, ERK1/2, and JNK) to activate the AP-1 transcription factor. Within the nucleus, NF-κB and AP-1 exhibit positive transcriptional synergy to upregulate pro-inflammatory mediators, driving two distinct pathological branches: the secretion of IL-1β, IL-6, and TNF-α, which mediates neutrophil chemotaxis within the PAMPs recognition network; the production of MMP12, leading to the early degradation of collagen fibers and extracellular matrix (ECM).

Pulpitis pathogenesis is driven by microbial invasion and the resulting disruption of microenvironmental homeostasis, which triggers aberrant intracellular signaling cascades (Figure 1). Current research suggests that the fate of pulp cells—whether progressing toward inflammatory necrosis or tissue repair—is primarily governed by the independent modulation of key signaling pathways and their multi-dimensional crosstalk.

### 2.1 TLR/NF-κB signaling axis-mediated early pathogen recognition and pro-inflammatory cascades

During the initial stage of pathogen invasion, TLRs serve as critical recognition receptors within the pulp’s innate immune system. PAMPs, such as lipopolysaccharide (LPS) from Gram-negative bacteria, are specifically recognized by TLR2 and TLR4 on the surface of pulp fibroblasts and macrophages (He F. et al., 2025). Upon activation, the intracellular adaptor protein MyD88 is recruited, initiating a downstream kinase cascade that leads to the phosphorylation and degradation of the inhibitory protein IκB. This, in turn, facilitates the nuclear translocation of the NF-κB p65-p50 dimer. Once in the nucleus, NF-κB regulates transcriptional activity, directly upregulating the expression of early pro-inflammatory cytokines such as IL-1β, IL-6, and TNF-α. In addition to mediating the release of inflammatory mediators, NF-κB can specifically bind to the promoter region of matrix metalloproteinase 12 (MMP12), activating its transcription and initiating the early degradation of the extracellular matrix, contributing to tissue damage (Xiao et al., 2025; Zhang X. et al., 2025). Furthermore, this immune recognition network is epigenetically modulated by endogenous non-coding RNAs. For example, long non-coding RNA (lncRNA) NUTM2A-AS1 acts as a competitive endogenous RNA by binding miR-769–5p, relieving its post-transcriptional inhibition of target genes, thereby enhancing the intensity of TLR4/MyD88/NF-κB signaling and exacerbating inflammatory damage to pulp cells (Li and Wang, 2025).

### 2.2 The regulatory role of the MAPK signaling pathway in the amplification of inflammatory mediators and hyperalgesia

The MAPK signaling network, comprising the p38, ERK1/2, and JNK subfamilies, plays a pivotal role in sensing pathogenic stress and orchestrating the inflammatory response in pulpitis. Upon LPS stimulation, human dental pulp cells (hDPCs) strongly activate the p38 MAPK and NF-κB pathways, driving the production of pro-inflammatory cytokines (Xiao et al., 2023). This inflammatory response is tightly regulated by intracellular transport mechanisms, such as the transport protein Importin7 (IPO7), which mediates the nuclear translocation of phosphorylated p38 (p-p38); silencing IPO7 significantly diminishes the inflammatory response (Xiao et al., 2023). Additionally, the MAPK pathway integrates signals from various local mediators, further amplifying inflammation. Wnt5a, which is upregulated in infected dental pulp, acts downstream of TNF-α to enhance the expression of key chemokines and cytokines, promoting macrophage recruitment in a MAPK- (p38, JNK, and ERK) and NF-κB-dependent manner (Zhao et al., 2014). In parallel with the cytokine storm, MAPK signaling also regulates pulpal vascular responses. LPS-induced expression of vascular endothelial growth factor (VEGF) in dental pulp fibroblasts and stem cells is critically dependent on the phosphorylation of ERK1/2 and PKC zeta, contributing to the pathological increase in microvascular density characteristic of pulpitis (Botero et al., 2010).

Beyond local tissue inflammation, the MAPK pathway is involved in peripheral sensitization and hyperalgesia, hallmark features of acute pulpitis. Tooth pain is primarily driven by sensitization of the trigeminal ganglion (TG), where ionotropic purinergic receptors, especially P2X3, play a central role in nociception (Chen et al., 2024b). Experimental models of irreversible pulpitis show that acute inflammation leads to a marked upregulation of P2X3, along with increased expression of p-p38, p-ERK1/2, TNF-α, and IL-1β within the TG (Chen et al., 2024a; Chen et al., 2024b). Activation of the P2X3 receptor triggers the downstream MAPK signaling cascade, perpetuating neurogenic inflammation and mediating significant allodynia and hyperalgesia in the maxillofacial region (Chen et al., 2024a). Importantly, selective P2X3 inhibitors, such as A-317491, suppress MAPK activation, reduce the release of inflammatory markers, and alleviate pain-like behaviors, making the P2X3-MAPK axis a promising therapeutic target for pulpitis-induced odontalgia (Chen et al., 2024a; Chen et al., 2024b). From a translational perspective, current analgesics like NSAIDs primarily target COX pathways and often fail to control this severe neurogenic hyperalgesia. While P2X3 antagonists offer a promising mechanism-based, non-opioid alternative, their systemic clinical translation is hindered by adverse effects such as dysgeusia. Therefore, developing responsive biomaterial carriers for localized, intra-pulpal delivery is crucial to maximize P2X3-targeted analgesic efficacy while minimizing systemic off-target effects.

Interestingly, while the MAPK pathway primarily drives inflammation and pain during acute infection, it exhibits pleiotropic effects depending on the microenvironmental context. During the regenerative phase, the activation of Erk1/2 and p38 MAPK is essential for TGF-β1-induced early odontoblastic differentiation of dental pulp stem cells (DPSCs), promoting the expression of key mineralization markers such as DMP-1 and Runx2 (Bai et al., 2023). In summary, MAPK signaling acts as a double-edged sword in pulpitis: it orchestrates acute inflammatory destruction and hyperalgesia during initial pathogen invasion, yet is subsequently repurposed to facilitate stem cell differentiation and matrix mineralization during tissue repair.

### 2.3 Roles and mechanisms of the HIF-1α signaling pathway in pulpitis

HIF-1α acts as a central transcriptional regulator within the pathological microenvironment of pulpitis. The dental pulp, enclosed within the rigid, low-compliance dentin chamber, rapidly develops a localized hypoxic environment during infection. This hypoxia results from the high metabolic oxygen consumption of infiltrating immune cells and bacteria, compounded by ischemia due to interstitial hypertension and LPS-induced microthrombosis (Jourdain et al., 2007; Lee et al., 2019). Under hypoxic conditions, the activity of prolyl hydroxylase domain (PHD) enzymes is inhibited, preventing the ubiquitin-mediated degradation of HIF-1α, thereby stabilizing it intracellularly and facilitating its nuclear translocation (Lee et al., 2020). Beyond hypoxia, LPS from Gram-negative bacteria can also directly upregulate HIF-1α expression at the transcriptional level by activating TAK1 kinase, which in turn activates the downstream NF-κB and p38 MAPK pathways (Li et al., 2014; van Uden et al., 2008).

During the acute inflammation phase of pulpitis, HIF-1α hypothesized to play a critical role in modulating the innate immune response through regulation of various cytokines. By enhancing the transcription of CD18, IL-1, and IL-8, HIF-1α significantly promotes neutrophil chemotaxis to the inflammatory site and stimulates the release of neutrophil extracellular traps (NETs) to bolster local bactericidal activity (Holder et al., 2019; Kong et al., 2004). Simultaneously, in conjunction with hypoxia and reactive oxygen species (ROS) signals, HIF-1α drives the polarization of monocytes into M1 pro-inflammatory macrophages, further intensifying the inflammatory cascade (Mills et al., 2016). While these classical immunological mechanisms provide a strong theoretical framework, their direct validation within the specific context of human pulpitis requires further investigation. Regarding cellular fate, HIF-1α promotes mitophagy of damaged mitochondria by upregulating BNIP3 and accelerates programmed cell death in the affected tissue by activating the NLRP3/ASC/Caspase-1 inflammasome through the NF-κB signaling axis in dental pulp fibroblasts (Wang D. et al., 2024; Wang X. et al., 2024).

As the inflammatory microenvironment transitions to the proliferation and remodeling stages, HIF-1α shifts its regulatory focus. Extrapolating from classical immunology studies, it is well-established that HIF-1α significantly enhances Th17 cell expansion by activating RORγt and upregulating glycolysis-related genes, while simultaneously inhibiting Treg cell development by promoting the degradation of the transcription factor FOXP3, thereby maintaining immune clearance during specific phases (Dang et al., 2011; Shi et al., 2011). Within the dental pulp-dentin complex specifically, HIF-1α has been shown to drive microvascular angiogenesis by binding to hypoxia-responsive elements (HREs) in the promoter regions of VEGF, FGF-2, and SDF-1 (Liu et al., 1995). Additionally, HIF-1α upregulates BMP-2 expression and induces the accumulation of the transcriptional cofactor BCL9, which synergizes with the Wnt/β-catenin pathway to promote the odontoblastic differentiation of DPSCs and the formation of reparative dentin bridges (Orikasa et al., 2022).

In summary, current evidence suggests that HIF-1α acts as an important contributor to the stage-dependent progression of pulpitis. While its specific roles in mediating local inflammasome activation and driving odontoblastic differentiation have been directly validated in pulpal models, its broader influence on immune cell polarization including M1 and Th17 shifts within the dentin chamber remains largely extrapolated from general immunology. Differentiating these systemic immunological paradigms from tissue-specific pulpal responses represents a critical frontier for future endodontic research (Shao et al., 2025).

### 2.4 Activation of the NLRP3 inflammasome and intersecting cascades of programmed cell death

Persistent hypoxia, oxidative stress, and the accumulation of metabolites significantly activate the NLRP3 inflammasome pathway in dental pulp cells. Numerous clinical studies have demonstrated a strong positive correlation between elevated NLRP3 expression in gingival crevicular fluid (GCF) and dental pulp tissue, along with specific gene polymorphisms, and the increased risk of irreversible pulpitis and the progression of periapical lesions (Neha et al., 2025; Verma et al., 2026).

At the molecular level, the pathological microenvironment acts as a key upstream signal driving inflammasome assembly. Under hypoxic conditions, dental pulp fibroblasts upregulate NLRP3 expression through the crosstalk between the HIF-1α and NF-κB signaling axes (Wang D. et al., 2024). Additionally, the extracellular accumulation of ATP activates the purinergic receptor P2X7 and related pathways, synergistically promoting NLRP3 inflammasome activation (Sun et al., 2023). Once activated, NLRP3 recruits the adaptor protein ASC and pro-Caspase-1, assembling a multiprotein complex that mediates the autocatalytic cleavage of Caspase-1.

Subsequently, activated Caspase-1 cleaves Gasdermin D (GSDMD), releasing its pore-forming N-terminal domain. This domain oligomerizes to form pores in the cell membrane, resulting in the efflux of cellular contents and mature pro-inflammatory cytokines IL-1β and IL-18, thereby triggering pyroptosis (Jiang et al., 2015; Wang D. et al., 2024). Furthermore, as an emerging concept in a severe inflammatory microenvironment, recent preliminary studies suggest that the ZBP1-NLRP3 signaling axis in dental pulp fibroblasts may play a role in integrating PANoptosis and ferroptosis networks. Although the evidence base within pulp biology is still in its early stages, the crosstalk between these programmed cell death pathways significantly contributes to driving dental pulp tissue toward irreversible liquefactive necrosis (He A. E. et al., 2025).

### 2.5 The roles of Wnt and TGF-β signaling pathways in pulpal inflammation and tissue remodeling

Despite the destructive inflammatory microenvironment, the dental pulp retains an intrinsic capacity for tissue repair and matrix remodeling, processes finely regulated by the Wnt and TGF-β signaling networks. During the progression of pulpitis, the Wnt signaling family exhibits context-dependent dual regulatory roles. On one hand, Wnt4 serves as a key protective factor, with its overexpression significantly reducing LPS-induced cellular apoptosis and inhibiting the release of pro-inflammatory cytokines by specifically targeting the phosphorylation of the IKK/NF-κB signaling axis (Ni et al., 2023). On the other hand, Wnt5a acts primarily as a pro-inflammatory mediator downstream of TNF-α. In the infected microenvironment, Wnt5a is markedly upregulated and utilizes the MAPK and NF-κB pathways to drive the secretion of inflammatory factors, including IL-17, which enhances macrophage chemotaxis and exacerbates localized immune infiltration (Liu et al., 2019; Zhao et al., 2014).

The TGF-β signaling pathway plays a central counter-regulatory role, bridging immune tolerance with structural regeneration (Smith, 2003). Clinical histological analyses confirm robust expression of TGF-β1 in the odontoblastic and subodontoblastic layers of irreversible pulpitis specimens, highlighting its critical role in initiating reparative dentinogenesis and stromal remodeling following severe injury (Piattelli et al., 2004). At the molecular defense level, odontoblasts—the first cells to encounter pathogen signals—use TGF-β1 to downregulate the expression of cell-surface microbial recognition receptors, such as TLR2 and TLR4, directly modulating the cellular response to invading bacteria (Horst et al., 2009). Ultimately, the dynamic balance between TLR-mediated pro-inflammatory networks and TGF-β-driven anti-inflammatory regenerative signals profoundly influences the tissue remodeling trajectory and the final fate of the inflamed dental pulp (Horst et al., 2009).

### 2.6 Dynamic signaling crosstalk across the spatiotemporal stages of pulpitis

The pathogenesis of irreversible pulpitis is not a collection of isolated molecular events but is driven by intricate, stage-dependent crosstalk between various signaling axes (Shao et al., 2025). The ultimate fate of the dental pulp, whether it progresses toward necrosis or undergoes repair, is determined by a continuous spatiotemporal tug-of-war between inflammatory, metabolic, and regenerative networks.

In the early stage of infection, pathogen recognition involves more than a single receptor pathway. TLR-mediated NF-κB activation and stress-induced MAPK signaling function synergistically to amplify the initial cytokine storm (Xiao et al., 2023). For instance, Wnt5a serves as a critical bridging mediator in this phase; it is upregulated by TNF-α and subsequently utilizes both MAPK and NF-κB pathways to drive macrophage chemotaxis and the secretion of IL-17 (Liu et al., 2019; Zhao et al., 2014). This initial crosstalk establishes a hyper-inflammatory focus that disrupts local homeostasis.

As inflammatory exudate increases interstitial pressure within the rigid dentin chamber, the resulting ischemia triggers a second wave of crosstalk, bridging metabolic reprogramming with programmed cell death. Under these hypoxic conditions, stabilized HIF-1α does not act in isolation but directly interacts with the NF-κB axis to transcriptionally upregulate NLRP3 expression in dental pulp fibroblasts (Wang D. et al., 2024). This metabolic-inflammatory convergence is further intensified by extracellular ATP, which activates P2X7 receptors to trigger NLRP3 inflammasome assembly and execute pyroptosis (Sun et al., 2023). Moreover, the ZBP1-NLRP3 signaling axis can integrate PANoptosis and ferroptosis networks, ultimately driving the tissue toward irreversible liquefactive necrosis (He A. E. et al., 2025).

During the transition to the structural repair phase, a profound antagonistic crosstalk occurs between destructive and regenerative networks. Endogenous TGF-β1 attempts to induce immune tolerance by actively downregulating the expression of microbial recognition receptors, such as TLR2 and TLR4, on odontoblasts (Horst et al., 2009). Conversely, the hyperactive pro-inflammatory environment—sustained by unchecked NF-κB and MAPK signaling—can robustly suppress the odontoblastic differentiation potential mediated by Wnt/β-catenin and TGF-β/Smad pathways (Ni et al., 2023; Zhao et al., 2014). For example, while Wnt4 possesses the capacity to inhibit the IKK/NF-κB axis to prevent apoptosis, this protective effect is often overwhelmed by persistent inflammatory signals in irreversible cases (Ni et al., 2023).

Therefore, decoding this crosstalk reveals that successful vital pulp therapy requires a holistic approach to reset the dynamic network—simultaneously dampening the destructive HIF-1α/NLRP3/NF-κB axis while unleashing the intrinsic Wnt/TGF-β regenerative potential.

## 3 Pharmacological interventions and biomaterial applications targeting core signaling pathways in pulpitis

**FIGURE 2:**
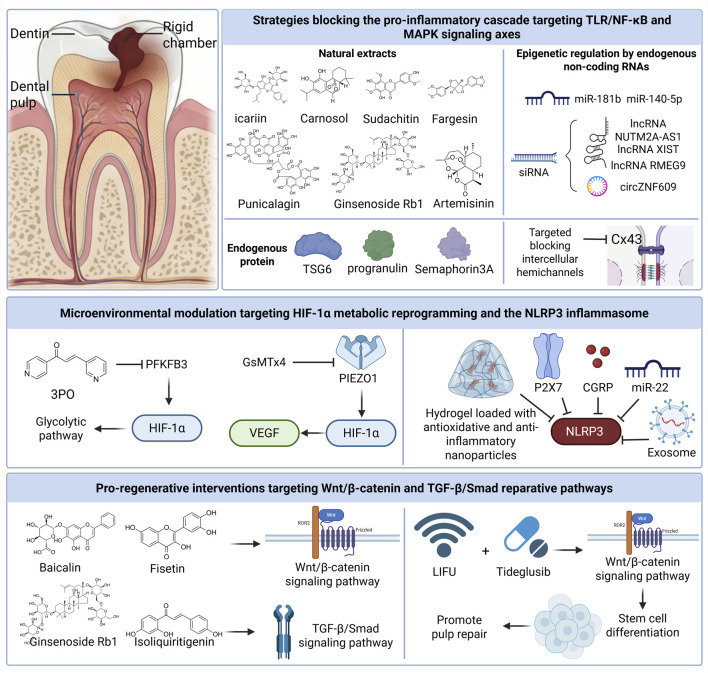
Overview of targeted pharmacological interventions and biomaterial applications in pulpitis. Based on the molecular mechanisms of pulpitis, current therapeutic strategies aim to shift the pathological microenvironment from inflammatory destruction to tissue repair by targeting core signaling networks. (1) Anti-inflammatory interventions: Natural small molecules and epigenetic tools selectively block the TLR/NF-κB and MAPK cascades to suppress cytokine storms. (2) Microenvironmental modulation: Specific inhibitors, smart hydrogels, and exosomes target HIF-1α metabolic reprogramming and the NLRP3 inflammasome to prevent pyroptosis and inflammatory cell death. (3) Pro-regenerative therapies: Novel biomaterialsand specific pharmacological agents synergistically activate the Wnt/β-catenin and TGF-β/Smad pathways, promoting the odontogenic differentiation of dental pulp stem cells (DPSCs) and reparative dentin formation.

Building on a deep understanding of the molecular network interactions during pulpitis progression, recent studies have focused on developing targeted intervention strategies to selectively inhibit pro-inflammatory cascades or activate endogenous reparative pathways. These strategies primarily include natural small-molecule compounds, epigenetic regulatory tools, endogenous pro-resolving mediators, and novel smart biomaterials (Table 1; Figure 2). However, it is crucial to interpret these promising pharmacological interventions with cautious optimism. A significant limitation of the current body of literature is the overwhelming reliance on *in vitro* cell culture models such as isolated human dental pulp cells and small animal models predominantly rodents. While these models are indispensable for elucidating basic mechanistic pathways, they inherently fail to recapitulate the complex polymicrobial biofilm challenges and the unique biomechanical constraints of the human low-compliance dentin chamber. Consequently, the robust anti-inflammatory and regenerative effects observed in these controlled experimental settings may not directly translate to the highly heterogeneous and mechanically stressed microenvironment of clinical irreversible pulpitis.

**TABLE 1:** Potential therapeutic interventions and molecular mechanisms targeting core signaling pathways in pulpitis.

Interventions	Targeted signaling pathways	Primary function	Mechanisms of action	Experimental models	References
Strategies blocking the pro-inflammatory cascade via tlr/NF-κb and MAPK					
Sudachitin (Natural polyphenol)	TLR2/NF-κB	Anti-inflammatory	Inhibits TLR2-mediated early receptor recognition, downregulating the transcription of downstream inflammatory mediators	*In vitro* (hDPCs), *In vivo* (Animal)	Mieda et al. (2025)
Carnosol	RAGE/NF-κB	Anti-inflammatory	Suppresses the RAGE/NF-κB signaling axis and nuclear translocation, mitigating localized cellular inflammatory stress	*In vitro* (hDPCs), *In vivo* (Animal)	Liu et al. (2025)
Icariin	TLR4/NF-κB	Anti-inflammatory	Downregulates TLR4-mediated immune recognition, attenuating the LPS-induced pro-inflammatory cascade	*In vitro* (hDPCs)	Liu et al. (2023)
Artemisinin	p53/NF-κB	Dual-function	Modulates cell proliferation and apoptotic networks, antagonizing pathogen-induced release of inflammatory mediators	*In vitro* (DPSCs), *In vivo* (Animal), Clinical	Sui et al. (2025)
Punicalagin	NF-κB/Wnt5a-ROR2	Dual-function	Suppresses NF-κB activation, mitigating inflammatory injury while alleviating the inhibition on tissue reparative differentiation	*In vivo* (Animal)	Yang et al. (2025)
Ginsenoside Rb1	PI3K/Akt, NF-κB, MAPK	Anti-inflammatory	Suppresses multiple kinase phosphorylations, antagonizing LPS-induced apoptosis and cytokine storms	*In vitro* (hDPCs), *In vivo* (Animal)	Nam et al. (2024)
Luteoloside	MAPK/NF-κB	Anti-inflammatory	Suppresses dual MAPK and NF-κB signaling pathways, attenuating methylglyoxal-induced inflammatory responses	*In vitro* (hDPCs), Clinical	Ji-Eun et al. (2024)
Fargesin	TLR4/NF-κB	Anti-inflammatory	Inhibits receptor signal transduction, mitigating apoptosis and localized oxidative stress	*In vivo* (Animal)	Song et al. (2022)
Dihydroquercetin	p38 MAPK	Anti-inflammatory	Regulates the p38 MAPK signaling pathway, attenuating cellular damage under inflammatory microenvironments	*In vitro* (hDPCs)	Kumagai et al. (2022)
Resveratrol	TLR/NF-κB, MAPK	Anti-inflammatory	Restricts IL-6 and IL-8 expression, suppressing the pro-inflammatory cascade triggered by LPS.	*In vitro* (hDPCs)	Paudel et al. (2014)
Luteolin	MAPK, Wnt/β-catenin	Dual-function	Suppresses the production of pro-inflammatory cytokines, preserving reparative potential while mitigating cellular stress	*In vitro* (hDPCs)	Zhao et al. (2014)
Epigallocatechin gallate (EGCG)	TLR/NF-κB, MAPK	Anti-inflammatory	Downregulates the expression of pro-inflammatory chemokines, antagonizing the host’s pro-inflammatory signaling network	*In vitro* (hDPCs)	Xiong et al. (2015)
MicroRNA-181 b introduction	PLAU/AKT/NF-κB	Anti-inflammatory	Regulates the pro-inflammatory signaling network by targeting PLAU, ameliorating the localized inflammatory microenvironment	*In vitro* (hDPCs), *In vivo* (Animal)	Meng et al. (2024)
MicroRNA-140–5p introduction	TLR4/NF-κB	Anti-inflammatory	Inhibits the TLR4 signaling axis, mitigating apoptosis and inflammation under pathological conditions	*In vitro* (hDPCs)	Zhai et al. (2020)
Knockdown of NUTM2A-AS1	TLR4/MYD88/NF-κB	Anti-inflammatory	Silences TLR4 via a ceRNA mechanism (releasing miR-769–5p), restricting downstream signal transduction and mitigating inflammatory injury	*In vitro* (hDPCs), *In vivo* (Animal)	Li and Wang (2025)
Knockdown of lncRNA XIST	miR-146a-5p/TRAF6	Anti-inflammatory	Releases targeted miRNAs to endogenously suppress the hyperactivation of the TRAF6/NF-κB signaling pathway	*In vitro* (hDPCs)	Kaewpitak et al. (2020)
Knockdown of circZNF609	miR-145–5p/TLR4	Anti-inflammatory	Blocks endogenous aberrant signaling axes, alleviating pathogen-induced apoptosis and the inflammatory cascade	*In vitro* (hDPCs)	Petrukhina et al. (2022)
Knockdown of lncRNA RMEG9	miR-125a-5p/p38 MAPK	Anti-inflammatory	Regulates the p38 MAPK signaling branch, mitigating programmed cell death and inflammatory damage	*In vivo* (Animal)	Zhai et al. (2019)
Connexin43 (Cx43) blockers	NF-κB	Anti-inflammatory	Interrupts the spatial propagation of NF-κB-dependent pro-inflammatory signals between adjacent cells via hemichannels, restricting tissue damage	*In vitro* (hDPCs), *In vivo* (Animal)	Long et al. (2024)
Recombinant TSG-6 protein	NF-κB	Dual-function	Feedback-inhibits the NF-κB pathway, preserving the odontoblastic differentiation potential of DPSCs while exerting anti-inflammatory effects	*In vitro* (DPSCs), *In vivo* (Animal)	Wang et al. (2024c)
Osteomodulin	IL-1R1/NF-κB	Dual-function	Regulates the NF-κB signaling cascade, exerting dual anti-inflammatory and tissue-protective effects in the pulpal microenvironment	*In vitro* (DPSCs), *In vivo* (Animal), Clinical	Yang et al. (2025)
Progranulin (PGRN)	TNFR2/14-3–3 epsilon/NF-κB	Dual-function	Suppresses NF-κB activation, facilitating anti-inflammatory macrophage polarization and pulpal tissue repair	*In vitro* (DPSCs, Macrophages), *In vivo* (Animal)	Ju et al. (2025)
Semaphorin3A	TLR4	Anti-inflammatory	Downregulates TLR4 receptor expression, suppressing localized immune hyperreactivity and cytotoxicity	*In vitro* (hDPCs)	Shindo et al. (2021)
Microenvironmental Modulation Targeting HIF-1α Metabolism and NLRP3 Inflammasome					
PFKFB3 Inhibitor (3PO)	HIF-1α/Glycolysis	Dual-function	Blocks HIF-1α-mediated excessive glycolysis, driving the microenvironment to transition from a pro-inflammatory to a tissue-reparative phenotype	*In vitro* (DPSCs, Macrophages), *In vivo* (Animal)	Zhao et al. (2025)
Ion channel inhibitor GsMTx4	PIEZO1/HIF-1α/VEGF	Anti-inflammatory	Blocks aberrant mechanotransduction and pathological metabolic signals, alleviating inflammation and oxidative stress in the localized microenvironment	*In vivo* (Animal)	Song et al. (2022)
Thermosensitive *in situ* hydrogel	NLRP3 Inflammasome/Pyroptosis	Anti-inflammatory	Modulates NLRP3 activity within the pathological microenvironment, suppressing pyroptotic and PANoptotic cell death cascades	*In vivo* (Animal)	Zhang et al. (2025a)
P2X7 receptor antagonists	NLRP3/Caspase-1	Anti-inflammatory	Antagonizes ligand-gated ion channels, blocking the assembly of the pyroptotic signaling axis and the activation of cell death programs	*In vivo* (Animal)	Sun et al. (2023)
Calcitonin gene-related peptide (CGRP)	NLRP3 Inflammasome	Anti-inflammatory	Inhibits NLRP3 inflammasome assembly, mitigating inflammatory programmed cell death in damaged dental pulp fibroblasts	*In vitro* (hDPCs)	Wang et al. (2021)
MicroRNA-22 introduction	NLRP3/CASP1	Anti-inflammatory	Suppresses the NLRP3-mediated inflammatory necrosis pathway, ameliorating hypoxia-induced cellular damage	*In vitro* (Fibroblasts), *In vivo* (Animal)	Jiang et al. (2022)
Intercellular mitochondrial transfer	NLRP3/Pyroptosis	Regenerative	Restores energy metabolism homeostasis in damaged cells via exosomes, alleviating oxidative stress-induced NLRP3 pyroptotic injury	*In vitro* (DPSCs), *In vivo* (Animal)	Wang et al. (2023)
Tissue regeneration targeting Wnt/β-catenin and TGF-β/smad reparative pathways					
Biodentine	Wnt/β-catenin	Regenerative	Activates this pathway, reversing the stem cell senescence process and preserving osteogenic/odontoblastic differentiation potential	*In vitro* (DPSCs), *In vivo* (Animal), Clinical	Zhang et al. (2025b)
Alkaline-treated dentin matrix extracts	Tissue regenerative networks	Regenerative	Releases endogenous growth factors, synergistically activating stem cell differentiation and pulpal tissue repair networks	*In vitro* (hDPCs, Macrophages), *In vivo* (Animal)	Jia et al. (2025)
Ginsenoside Rb1	TGF-β/Smad	Regenerative	Upregulates reparative pathways, promoting DPSC differentiation and the mineralization/deposition of reparative dentin	*In vitro* (DPSCs), *In vivo* (Animal)	Nam et al. (2026)
Isoliquiritigenin	TGF-β/Smad	Regenerative	Activates the TGF-β/Smad signaling pathway, facilitating the odontoblastic differentiation efficacy of stem cells	*In vitro* (DPSCs)	Hu et al. (2021)
Baicalin	Wnt/β-catenin	Regenerative	Activates the canonical Wnt pathway, enhancing tissue remodeling and differentiation efficacy of stem cells under inflammatory microenvironments	*In vitro* (DPSCs), *In vivo* (Animal)	Li et al. (2023)
Fisetin	Wnt/β-catenin	Regenerative	Activates Wnt/β-catenin signaling, promoting the repair of damaged tissues while alleviating localized oxidative stress	*In vivo* (Animal)	Lei et al. (2022)
LIFU + Tideglusib	Wnt/β-catenin, TGF-β/Smad	Regenerative	Synergizes physical mechanostimulation (Low-intensity focused ultrasound) with chemical targeted agonists to enhance the tissue remodeling and mineralization efficacy of stem cells	*In vitro* (DPSCs), *In vivo* (Animal)	Maria et al. (2025)

### 3.1 Strategies blocking the pro-inflammatory cascade targeting TLR/NF-κB and MAPK signaling axes

Given the central role of the TLR/NF-κB and MAPK pathways in initiating and amplifying inflammation, suppressing their hyperactivation has become a key pharmacological approach for controlling pulpal inflammatory injury. Extensive *in vitro* and animal model studies have shown that highly biocompatible phytochemicals—especially polyphenols, flavonoids, and terpenoids—can selectively block these pathways at multiple levels. Nevertheless, from a translational perspective, the clinical utility of these phytochemicals remains largely theoretical. Critical issues such as optimal therapeutic dosing, local bioavailability within the ischemic pulp tissue, and long-term stability in the acidic inflammatory exudate are rarely addressed in these preliminary studies. Furthermore, many *in vitro* assays apply these compounds prophylactically or immediately alongside LPS stimulation, which does not accurately reflect the delayed clinical presentation of advanced pulpitis. For example, natural extracts such as sudachitin, carnosol, icariin, and artemisinin significantly downregulate inflammatory mediator transcription by specifically antagonizing TLR-mediated early signaling (Liu et al., 2023; Liu et al., 2025; Mieda et al., 2025; Sui et al., 2025). Additionally, various bioactive molecules, including punicalagin, ginsenoside Rb1, flavonoid mixtures, luteoloside, fargesin, and dihydroquercetin, achieve multi-target interventions by inhibiting PI3K/Akt, NF-κB nuclear translocation, and MAPK hyperphosphorylation, thus broadly mitigating LPS-induced apoptosis and pro-inflammatory cytokine storms (Ji-Eun et al., 2024; Kumagai et al., 2022; Mendes Soares et al., 2024; Nam et al., 2024; Song et al., 2022; Yang et al., 2025). Furthermore, classical literature highlights that resveratrol, luteolin, and epigallocatechin gallate (EGCG) exert significant negative regulatory effects on the MAPK pathway in pulpitis (Guo and Chen, 2019; Paudel et al., 2014; Xiong et al., 2015; Zhao et al., 2014). Despite these promising results, the clinical translation of phytochemicals is currently constrained by their inherently poor bioavailability and the lack of human pharmacokinetic data. Most existing evidence is derived from *in vitro* 2D models, which do not fully replicate the complex metabolic challenges and acidic microenvironment of the inflamed pulp chamber. Consequently, the lack of randomized clinical trials and standardized dosing regimens underscores the need for more rigorous translational validation before these natural compounds can be implemented in precision endodontics.

At the level of post-transcriptional and intercellular communication regulation, targeting epigenetic networks holds great potential for precise intervention. The exogenous delivery of protective microRNAs, such as miR-181b and miR-140–5p, or the use of siRNAs to knock down specific lncRNAs and circular RNAs (circRNAs), including NUTM2A-AS1, lncRNA XIST, circZNF609, and lncRNA RMEG9, can effectively downregulate the TLR4/MYD88/NF-κB signaling axis, reducing DNA damage and tissue destruction in dental pulp cells (Kaewpitak et al., 2020; Li and Wang, 2025; Meng et al., 2024; Petrukhina et al., 2022; Zhai et al., 2019; Zhai et al., 2020). However, it is crucial to recognize that the clinical translation of these epigenetic tools faces formidable challenges. Naked non-coding RNAs suffer from inherent molecular instability and rapid enzymatic degradation within the highly acidic inflammatory exudate of the pulp chamber. Furthermore, achieving targeted delivery efficiency into specific pulp cells without triggering unintended off-target genetic silencing remains a major pharmacological obstacle. Addressing these technical barriers, alongside navigating the stringent regulatory concerns associated with nucleic acid-based therapeutics, is imperative before these promising epigenetic interventions can be considered clinically viable. Moreover, blocking Connexin43 (Cx43)-mediated intercellular hemichannels effectively disrupts the spatial propagation of NF-κB-dependent pro-inflammatory signals between adjacent cells, limiting lesion expansion (Long et al., 2024). Additionally, recombinant proteins such as TSG-6, osteomodulin, progranulin, and Semaphorin3A have demonstrated significant anti-inflammatory and pulp-protective effects by feedback-inhibiting the NF-κB pathway (Ju et al., 2025; Shindo et al., 2021; Wang Y. et al., 2024; Yang et al., 2025).

Importantly, the therapeutic paradigm for pulpitis is shifting from passive inflammatory suppression to active ‘resolution pharmacology' driven by specialized pro-resolving mediators (SPMs) (Serhan, 2014). Unlike traditional agents that merely block pro-inflammatory cascades, Resolvin E1 (RvE1) actively restores tissue homeostasis. Mechanistically, RvE1 engages ChemR23 receptors to attenuate neutrophil infiltration, enhance macrophage efferocytosis, and suppress NF-κB signaling (Serhan et al., 2015). Translationally, incorporating RvE1 into biomaterial scaffolds can dynamically reprogram the inflamed microenvironment, directly bridging immune resolution with Axin2+ stem cell-mediated reparative dentinogenesis (Wu et al., 2023).

### 3.2 Microenvironmental modulation targeting HIF-1α metabolic reprogramming and the NLRP3 inflammasome

In response to the severely ischemic, hypoxic, and metabolically dysregulated microenvironment of the dental pulp, emerging intervention strategies are increasingly focused on reversing metabolic reprogramming and preventing inflammatory cell death. Regarding metabolic interventions, the use of the specific PFKFB3 inhibitor (3PO) to block the HIF-1α-mediated excessive glycolytic pathway can significantly alter the energy metabolism of dental pulp cells, shifting the localized microenvironment from a pro-inflammatory to a tissue-reparative phenotype (Zhao et al., 2025). Additionally, the mechanosensitive ion channel inhibitor GsMTx4 has been shown to effectively alleviate localized inflammation and oxidative stress in a rat pulpitis model by blocking PIEZO1/HIF-1α/VEGF signaling (Song et al., 2022).

To counteract pyroptosis and PANoptosis mediated by the NLRP3 inflammasome, novel delivery systems and targeted therapies have been developed. A pioneering study in materials science introduced a thermosensitive injectable *in situ* hydrogel loaded with antioxidative and anti-inflammatory nanoparticles. This smart material can elp to downregulate NLRP3 activity within the pathological microenvironment, thereby mitigating programmed cell death cascades triggered by environmental stress in VPT applications (Zhang L. et al., 2025). Additionally, antagonizing the ligand-gated ion channel P2X7, or applying calcitonin gene-related peptide (CGRP) and specific microRNAs (e.g., miR-22), has been shown to block the assembly and activation of the NLRP3/Caspase-1 axis, effectively mitigating inflammatory death in dental pulp fibroblasts (Jiang et al., 2022; Sun et al., 2023; Wang et al., 2021). Moreover, exosomes derived from DPSCs and exosome-mediated mitochondrial transfer technologies have been shown to alleviate NLRP3-mediated pyroptotic injury by restoring energy metabolic homeostasis in damaged target cells, offering novel insights for cell-free regenerative therapies (He et al., 2021; Wang et al., 2023).

### 3.3 Pro-regenerative interventions targeting Wnt/β-catenin and TGF-β/smad reparative pathways

Once the inflammatory microenvironment is controlled, activating DPSCs and remodeling the damaged extracellular matrix are critical for successful VPT. Modern pulp capping materials, particularly calcium silicate-based cements (CSCs) such as Biodentine, primarily activate endogenous regenerative signaling pathways. CSCs potently activate the Wnt/β-catenin signaling pathway, which reverses the cellular senescence of DPSCs under inflammatory conditions and preserves their osteogenic and odontogenic differentiation potential, particularly within specialized Axin2+ stem cell populations that are essential for targeted reparative dentinogenesis (Chung et al., 2019; Zhang Q. et al., 2025). Similarly, alkaline-treated dentin matrix extracts can further activate the reparative network by releasing multiple growth factors (Jia et al., 2025).

In addition to inorganic biomaterials, specific pharmacological small molecules exhibit high pathway specificity in promoting regeneration. Monomers from traditional Chinese medicine, such as ginsenoside Rb1 and isoliquiritigenin, significantly enhance the odontogenic differentiation of DPSCs and mineralized dentin deposition by upregulating the TGF-β/Smad signaling pathway (Hu et al., 2021; Nam et al., 2026). Baicalin and fisetin also promote tissue remodeling by activating the Wnt/β-catenin pathway (Lei et al., 2022; Li et al., 2023). Furthermore, combining low-intensity focused ultrasound (LIFU) with the Wnt signaling agonist Tideglusib represents a cutting-edge regenerative paradigm, where physical mechanostimulation and chemical signaling synergistically activate stem cell differentiation networks (Maria Rajan et al., 2025).

## 4 Conclusion and perspectives

The pathogenesis of pulpitis is not driven by isolated inflammatory mediators but rather by a highly interconnected signaling network that includes TLR/NF-κB-mediated immune activation, HIF-1α-driven metabolic stress, NLRP3-induced pyroptosis, and Wnt/TGF-β-regulated tissue remodeling. While significant advances have been made in understanding the roles of individual pathways and their targeted interventions, achieving the clinical goals of objective diagnosis and predictable VPT for irreversible pulpitis requires further investigation in three key areas.

### 4.1 Signaling crosstalk and multi-targeted interventions

First, current mechanistic studies predominantly focus on linear elucidation of isolated pathways, often neglecting the complex crosstalk within the pathological microenvironment. In actual inflammatory lesions, hyperactivated pro-inflammatory signals, particularly those through NF-κB and MAPK, often antagonize and suppress the regenerative Wnt/β-catenin and TGF-β/Smad networks. Therefore, future research should prioritize exploring the mutual regulatory mechanisms between pro-inflammatory and reparative cascades. From a pharmacological perspective, the development of novel therapeutics should aim at dual immunomodulation. For instance, multi-target agents, such as specialized pro-resolving lipid mediators like Resolvins, show great potential in simultaneously dampening pro-inflammatory pathways and alleviating the inflammatory suppression of cellular differentiation, thereby promoting the structural repair of the dentin-pulp complex (Chen et al., 2021; Liu et al., 2022; Wu et al., 2023). Furthermore, accelerating the translation of preclinical discoveries into clinically viable therapies necessitates a rigorous reevaluation of experimental models. Future research must move beyond conventional 2D monocultures toward physiologically faithful systems, such as 3D dental pulp organoids or tooth-on-a-chip platforms, that accurately reproduce critical features of the human pulp microenvironment, including dynamic hemodynamic flow, interstitial pressure gradients, and multicellular cellular crosstalk. Equally critical is the initiation of large-scale, prospectively designed, and rigorously controlled human clinical trials to establish the therapeutic efficacy, safety profile, and clinical relevance of these targeted molecular interventions. Only evidence derived from such high-standard clinical investigations, not inference from proof-of-concept animal studies, can reliably inform regulatory approval, clinical adoption, and evidence-based decision-making in regenerative endodontics.

### 4.2 Biomarker discovery and clinical diagnostic translation clinically

Second, the differentiation of reversible from irreversible pulpitis still relies heavily on subjective pain history and traditional thermal or electrical sensibility tests, which lack objective criteria reflecting the true pathological status of the pulp. With our expanded understanding of pulpal signaling networks, translational medicine must pursue objective diagnostic technologies based on molecular biomarkers. Upregulated NLRP3 inflammasome activity, cGAS-STING signaling molecules, or specific non-coding RNAs detected in GCF or minimally invasively sampled pulpal blood could serve as reliable indicators of inflammatory severity (Tian et al., 2022; Verma et al., 2026). The development of non-invasive point-of-care testing (POCT) technologies targeting these pathways could optimize clinical decision-making, providing a solid molecular rationale for selecting between VPT and root canal treatment. However, the clinical translation of these objective diagnostics must overcome challenges regarding biomarker reproducibility, as sampling minute volumes of pulpal blood or gingival crevicular fluid is highly susceptible to contamination, necessitating standardized protocols and large-cohort validation.

### 4.3 Single-cell multi-omics and cellular heterogeneity

Third, contemporary pulp biology increasingly recognizes that “dental pulp cells” do not function as a unified monolith. Instead, signaling crosstalk is orchestrated by a highly heterogeneous network of specialized cell populations. Due to the pronounced spatial and physicochemical heterogeneity within the enclosed pulp chamber—characterized by severe hypoxia in the coronal pulp and normoxia in the radicular pulp—the integration of single-cell RNA sequencing (scRNA-seq) and spatial transcriptomics into mechanistic studies is essential. Multi-omic analyses will allow for precise, single-cell resolution characterization of distinct macrophage subsets, fibroblast heterogeneity, endothelial cells mediating localized angiogenesis, and peripheral nerve-related Schwann cell-associated progenitors. Decoding how these specific subpopulations interact across distinct anatomical regions will provide unprecedented insights into the transcriptional differences in metabolic reprogramming and inflammatory resolution (Wu et al., 2025).

Finally, the clinical translation of biomaterials requires next-generation pulp capping agents and regenerative scaffolds to have microenvironment-responsive properties. Ideal bioactive materials should act as smart sensors, detecting pathological physicochemical changes such as the mildly acidic environment produced by HIF-1α-mediated glycolysis or the localized accumulation of ROS from NLRP3 activation. These materials could then trigger the targeted release of kinase inhibitors or transforming growth factors. Such responsive material systems, capable of dynamically adapting to the fluctuating pathological microenvironment, will provide essential material science support for successful VPT under complex infectious conditions (Zhang L. et al., 2025).

Despite these conceptually attractive advances, the integration of biomarker-guided precision therapeutics and dynamic microenvironment-responsive scaffolds must be viewed cautiously as long-term future directions rather than near-term clinical realities. Transitioning these innovations from the bench to the chairside requires overcoming profound clinical hurdles. Specifically, a critical discrepancy remains between experimental models and human clinical pulpitis, as signaling pathways often behave differently depending on local oxygen tension, immune composition, and tissue architecture. The unique low-compliance dentin chamber severely restricts local drug delivery and diffusion; moreover, bridging the gap between controlled *in vitro*/animal systems and polymicrobial human infections requires rigorous safety and regulatory evaluations, ultimately demanding validation through well-designed randomized clinical trials.
